# Micro Freeze-Dryer and Infrared-Based PAT: Novel Tools for Primary Drying Design Space Determination of Freeze-Drying Processes

**DOI:** 10.1007/s11095-021-03023-x

**Published:** 2021-03-08

**Authors:** Maitê Harguindeguy, Davide Fissore

**Affiliations:** Dipartimento di Scienza Applicata e Tecnologia, Politecnico di Torino, Corso Duca degli Abruzzi 24, 10129 Torino, Italy

**Keywords:** Design space, primary drying, freeze-drying process design/optimization, heat and mass transfer, mechanistic approach, model parameters

## Abstract

**Purpose:**

Present (i) an infrared (IR)-based Process Analytical Technology (PAT) installed in a lab-scale freeze-dryer and (ii) a micro freeze-dryer (MicroFD®) as effective tools for freeze-drying design space calculation of the primary drying stage.

**Methods:**

The case studies investigated are the freeze-drying of a crystalline (5% mannitol) and of an amorphous (5% sucrose) solution processed in 6R vials. The heat (*K*_*v*_) and the mass (*R*_*p*_) transfer coefficients were estimated: tests at 8, 13 and 26 Pa were carried out to assess the chamber pressure effect on *K*_*v*_. The design space of the primary drying stage was calculated using these parameters and a well-established model-based approach. The results obtained using the proposed tools were compared to the ones in case *K*_*v*_ and *R*_*p*_ were estimated in a lab-scale unit through gravimetric tests and a thermocouple-based method, respectively.

**Results:**

The IR-based method allows a non-gravimetric estimation of the *K*_*v*_ values while with the micro freeze-dryer gravimetric tests require a very small number of vials. In both cases, the obtained values of *K*_*v*_ and *R*_*p*_, as well as the resulting design spaces, were all in very good agreement with those obtained in a lab-scale unit through the gravimetric tests (*K*_*v*_) and the thermocouple-based method (*R*_*p*_).

**Conclusions:**

The proposed tools can be effectively used for design space calculation in substitution of other well-spread methods. Their advantages are mainly the less laborious *K*_*v*_ estimation process and, as far as the MicroFD® is concerned, the possibility of saving time and formulation material when evaluating *R*_*p*_.

## Introduction

Freeze drying is a process widely used in the pharmaceutical industry to recover drug formulations from aqueous solutions. The liquid product is usually poured into vials, loaded in the freeze dryer where the process is carried out. First the solution is frozen, then the pressure is lowered, and heat is supplied to promote sublimation of the solvent (primary drying). Finally, the unfrozen water present in the product cake is removed by further increasing the temperature of the product (secondary drying) (1).

Freeze drying is a long and costly process, and is generally used over other drying methods when the drug formulation is heat sensitive (2). Notwithstanding the price, it is estimated that 16% of the top-selling 100 pharmaceuticals are freeze-dried (3). Therefore, is it imperative to have fast and efficient freeze-drying process development (and optimization) tools, shortening the time-to-market and providing benefits to the patients.

During primary drying, it is a good practice to keep product temperature below the critical temperature of the formulation, which usually is the glass transition temperature (*T*_*g*_) for amorphous systems or the eutectic point (*T*_*e*_) for crystalline ones. If this threshold is surpassed, changes in the cake porous structure may jeopardize product solubility, drug activity and overall product quality (4). In some cases such as nanoparticle suspensions (5), highly concentrated proteins (6) or the combined used of crystalline and amorphous bulking agents, surpassing the critical temperature may present macrocollapse, while not necessarily affecting product quality (7). Nonetheless, it is of crucial importance to identify and use the correct operating conditions that will preserve product quality.

The set of operating conditions that ensure product temperature to be below its threshold value for a given batch configuration is the design space. These operating conditions are the chamber pressure (*P*_*C*_) and the shelf temperature (*T*_*shelf*_) settings, the former defines the vapor pressure that must be achieved for sublimation to occur and the later provides the heat for sublimation. To minimize the time-to-market for a given product, it is therefore necessary to quickly identify a suitable couple of values of *P*_*C*_ and *T*_*shelf*_ that allow obtaining the target quality in the final product. Besides, it must be considered that primary drying alone was shown to represent 69% of the operational costs in an industrial freeze-dryer. However, the operational costs represent less than 15% of the total costs, which includes capital ones. Withal, shortening freeze-drying cycle durations increases productivity which in turn reduces the capital costs per cycle (8). To optimize the process further, the settings that maximize the sublimation rate are preferred because they make the cycle faster (9). However, solvent flow rate must be compatible with the freeze-dryer condenser capacity and also with the features of the duct connecting the chamber to the condenser, to avoid choked flow (10–12). Thus, these optimal settings must be carefully selected within the design space.

To obtain the design space, empirical and mechanistic approaches can be used. An empirical approach can be performed by a non-expert practitioner, but it requires many time-consuming experiments to determine the relationship between the operating conditions and the resulting process. Besides, this approach is only valid in situ, which limits the scalability of the results found at lab-scale, where these experiments may be carried out (13, 14). Mechanistic approaches, on the other hand, allow mathematical modelling of the product temperature, water vapor flow and drying time throughout a process as a function of the chamber pressure and shelf temperature (15–18). Such models are based on heat and mass transfer balances and can be used once parameters like the global heat transfer coefficient (*K*_*v*_) and the cake resistance to vapor flow (*R*_*p*_) are known. When this approach is used, fewer experiments are needed with respect to the empirical approach to obtain a comprehensive design space for a product. Nonetheless, even when using a mechanistic approach, the design space determination for a formulation is a time-consuming task.

The gravimetric method (15, 19, 20) is regarded as the standard method to obtain *K*_*v*_; however, many alternative methods have been proposed. In general terms, if reliable product temperature monitoring and primary drying end-point determination tools are in place, non-gravimetric *K*_*v*_ estimations can be obtained. Many of the alternative methods are based on the pressure rise test (PRT) using different algorithms, varying in complexity. Some of this methods are the Pressure Rise Analysis (PRA) (21), the Manometric Temperature Measurement (MTM) (22), Dynamic Parameters Estimation Method (DPE) (2) and its more straightforward version DPE+ (23). Other methods presented were based on a heat flux sensor (24) and Tuneable Diode Laser Absorption Spectroscopy (TDLAS) (25–27) to cite a few. Some advantages can be obtained with these methods, for instance, the determination of *K*_*v*_ at different pressures in the same run using a heat-flux sensor. The main drawback is that a mean value of *K*_*v*_ is obtained for the batch, without differentiating between the central vials, heated just through the shelf, and the edge vials, heated also through other mechanisms, e.g. radiation from chamber walls and door (19), while gravimetric test provides a very detailed picture of the system. As far as *R*_*p*_ is concerned, it may be estimated as well as by means of PRT-based algorithms (23, 28), through TDLAS (29), or using the product temperature measurement in a run (30).

In this study, we present two methods for design space determination, based on the following devices:

i. An Infrared (IR) process analytical technology (PAT) tool for monitoring a lab-scale freeze-dryer to obtain *R*_*p*_ and a non-gravimetric *K*_*v*_ estimation in a non-invasive way, i.e., without using thermocouples (to the authors’ knowledge, this is the first time this has been successfully implemented using this type of sensor).
ii. A micro freeze-dryer, to obtain the model parameters *R*_*p*_ and *K*_*v*_ using fewer vials than usual.

As a base for comparison, the standardly used tool for mechanistic approaches, a freeze-dryer equipped with thermocouples, is also presented. The model parameters and design spaces obtained through the innovative methods are compared to the ones obtained using the standard methods for verification of their applicability. The design spaces for central batch conditions for two different systems, an amorphous and a crystalline one, are tested. Central batch conditions are those applicable to central vials in a batch, i.e., those with at least 6 neighbouring vials. Central vials typically correspond to more than 90% of the vials in industrial batch processes. Thus, the design spaces for edge vials, those with 5 of less neighbouring vials, are not presented in this study. The advantages and limitations of each novel approach are also discussed throughout this study.

## Materials & Methods

### Equipment

The experiments were carried out in two freeze dryers, a lab-scale one (REVO®) and a small scale one (MicroFD®), both produced by Millrock Technology Inc. (Kingston, NY, USA). The shelf temperature can be set from −70°C to +65°C in the REVO® and from −60°C to +60°C in the MicroFD® freeze-dryer. The REVO® has roughly 1 m^2^ of total shelf area and it is provided with an external condenser with maximum condensing capacity of 30 L operated at approximately −80°C. The MicroFD® has a chamber with a 15-cm-diameter circular shelf where the vials are loaded, encircled by removable thermal conductors. These conductors ensure the contact between the external vials of the batch and the temperature-controlled aluminium ring (LyoSim®). The LyoSim® is used to emulate the desired heating conditions observed in a larger batch, whether for edge or central batch conditions. To this end, the ring temperature can be set to range from −15°C to +15°C offset with respect to the average product temperature.

Chamber pressure was monitored in both freeze-dryers using a thermal conductivity (Pirani type) and a capacitive (Baratron type) pressure gauge. The ratio between these two pressure signals (*Pi*/*Ba*) was used to estimate the duration of the primary drying stage. The pressure profile by the Pirani gauge exhibits a sharp decreasing trend as the drying process comes to an end. The start of this inflection is defined as the onset time while the end of it is defined as the offset time (4). The time interval between these two points can be used to infer batch heterogeneity, while the drying duration lays, typically, between them. This is a broadly used method, while the use of the offset point to determine the end point is a good practice to ensure drying of all vials (31, 32). Both systems were equipped with T-type thermocouples (Tersid, Milano, Italy) for temperature monitoring. Additionally, an infrared sensor was used to monitor product temperature, when applicable.

The IR sensor used in this study (IMC Service S.r.l., Italy) is the same sensor presented by Harguindeguy & Fissore (33) to monitor batches also using the REVO® freeze-dryer. This system has a built-in thermal camera (FLIR Systems model A35; FLIR Systems Inc., Wilsonville, OR, USA), a processing board, and a Wi-Fi antenna for wireless data transfer. The IR sensor was placed inside the chamber, 25 cm away from the vials being monitored and on the same shelf. It was aligned with the shelf centreline, against the rear of the chamber. Placing the sensor in this way allows monitoring the whole cake axial profile, from the cake bottom to the top. The sublimation interface temperature is measured and tracked as the minimum axial temperature. The bottom temperature is the average temperature at the bottom acquisition pixels, both computed as previously described (33).

### Model Parameters

One-dimensional models, assuming negligible temperature and composition gradients in the radial direction of the vial, are able to well represent product temperature dynamics (34). They assume that the heat flux to the product is proportional to the temperature difference between the shelf temperature and the temperature of the product at the bottom of the vial (*T*_*b*_):

1 $$ {J}_q={K}_v\left({T}_{shelf}-{T}_b\right). $$

The water vapor mass flux from the sublimation interface to the drying chamber is proportional to the difference between their water vapour partial pressures, where the chamber water partial pressure can be assumed to be equal to the chamber pressure (*P*_*c*_) as the gas in the chamber is about 100% water vapor:

2 $$ {J}_w={\frac{1}{R}}_p\left({p}_{w,i}-{p}_{w,c}\right), $$

*p*_*w*, *i*_ may be calculated by Eq. 3, where *T*_*i*_ can be approximated to *T*_*b*_ when the cake height and *R*_*p*_ are low. In the present study these differences were smaller than 1°C during primary drying, as experimentally measured by the IR sensor.

3 $$ {p}_{w,i}={e}^{\left(28.935-\frac{6150}{Ti}\right)}. $$

The one-dimensional model used here is based on the energy balance at the sublimation interface (34):

4 $$ {J}_q=\Delta  {H}_s{J}_w, $$

stating that all the heat arriving to the interface of sublimation is used for ice sublimation. This equation (Eq. 4) may be used once *K*_*v*_ and *R*_*p*_ are known. With respect to the overall heat transfer coefficient *K*_*v*_, a gravimetric test may be carried out as described by many in the literature (16, 24, 35). In such tests, the total heat received by the vials (*Q*) is assumed to be used for water sublimation, quantified by the weight loss (∆*m*) in each vial after a truncated sublimation cycle:

5 $$ Q=\Delta  m\Delta  {H}_s. $$

The amount of heat received by the product can be also expressed as:

6 $$ Q={K}_v{A}_v{\int}_0^{t_d}\left({T}_{shelf}-{T}_b\right) dt, $$

where *t*_*d*_ is the duration of the sublimation step of the gravimetric test and *A*_*v*_ is the cross-section area of the vial. With Eq. 5 and Eq. 6, it is possible to determine *K*_*v*_ if *T*_*shelf*_ and *T*_*b*_ are known. The global heat exchange coefficient, *K*_*v*_, may be also obtained at the end of a full primary drying cycle given that also the drying end-time is accurately determined. In fact, at the end of the drying process, ∆*m* corresponds to the initial amount of water in each vial, and Eq. 6 may be used to get *K*_*v*_ by setting *t*_*d*_ equal to the duration of the primary drying stage.

The heat exchange coefficient depends mainly on the type of vial used and on the chamber pressure, whereas the heating fluid temperature has a negligible effect (19). This way, *K*_*v*_ can be estimated as a function of pressure for a given product-vial set up (36) as illustrated in Eq. 7:

7 $$ {K}_v={a}_{K_v}+\frac{b_{K_v}{P}_c}{1+{c}_{K_v}{P}_c}. $$

The *K*_*v*_ fit coefficients *b*_*Kv*_, *c*_*Kv*_ give the dependence of *K*_*v*_ on *P*_*c*_ and their dependence on the equipment can be neglected. The coefficient *a*_*Kv*_, on the other hand, has a high dependence on the equipment and on the position of the vial over the shelf (37).

To obtain *R*_*p*_, first the *K*_*v*_ for that batch configuration and settings must be known. Then, product temperature must be monitored for the studied formulation during a drying cycle (where the operating conditions are set in such a way that cake collapse is avoided). Using Eq. 1*J*_*q*_ is obtained to then obtain *J*_*w*_ through Eq. 4. Since *p*_*w*, *i*_ is a function of product temperature and *p*_*w*, *c*_ can be assumed to be equal to *P*_*c*_, *R*_*p*_ can be obtained using Eq. 2.

*R*_*p*_ can be described in function of the dried cake thickness (*L*_*dried*_), which can be calculated based on the water mass flux (*J*_*w*_) (28). To account for this dependence between *R*_*p*_ and *L*_*dried*_, Eq. 8 is frequently used (36, 38, 39).

8 $$ {R}_p={R}_{p,0}+\frac{A{L}_{dried}}{1+B{L}_{dried}}. $$

In this model, *R*_*p*, 0_, *A* and *B* are fitted experimentally based on the *R*_*p*_ vs. *L*_*dried*_ values.

To simulate in silico the process as it progresses and calculate *T*_*b*_ according to how much frozen cake is still left, Eq. 9, i.e., the steady-state heat balance for the frozen product, can be used:

9 $$ {T}_b={T}_{shelf}-{\frac{1}{K}}_v{\left({\frac{1}{K}}_v+\frac{L_{frozen}}{k_{frozen}}\right)}^{-1}\left({T}_{shelf}-{T}_i\right). $$

The sublimation interface temperature, *T*_*i*_, is calculated recursively together with *p*_*w*, *i*_ and *R*_*p*_, using Eq. 2–4 and Eq. 8–9 for each integration interval. Twenty-second intervals were used in this simulation. Once *T*_*i*_ is found, *T*_*b*_ can be calculated for any stage of freeze drying, i.e., for any given percentage of frozen cake left. In Eq. 9, *k*_*frozen*_ is the ice conductivity. The *k*_*frozen*_ value used was, 2.55 *W*/*mK* (40), corresponding to the ice conductivity at −35°C.

### Design Space

For design space estimation using a mechanistic approach, the model parameter *K*_*v*_ must first be determined as a function of chamber pressure, as presented in Eq. 7. To this end, at least three gravimetric tests should be carried out at different pressures as described in Fissore et al (36). These gravimetric tests can be performed with water to save formulation material and preparation time as the solution composition has no effect on the resulting *K*_*v*_ (15).

For *R*_*p*_ estimation, at least one complete primary drying cycle should be performed for the target formulation. It is important to ensure that product temperature during this test is kept below the threshold value for that product. Otherwise, cake collapse takes place, leading to misestimation of the *R*_*p*_ profile. If this happens, product temperature during primary drying will be also misestimated and the resulting design space will not ensure product quality (14).

Having these parameters properly computed, the *T*_*shelf*_ and *P*_*c*_ combinations that will ensure product *T*_*b*_ to be below the formulation threshold value can be calculated. Eq. 1–4 can be used to this end, determining the possible *T*_*shelf*_ and *P*_*c*_ combinations for each and any point of primary drying progress, defined by the residual *L*_*frozen*_. This way, for each pressure value being considered in the design space, the product *T*_*b*_ for the regarded *L*_*frozen*_ is calculated by testing different *T*_*shelf*_ values. Thus, the *T*_*shelf*_ values that ensure product *T*_*b*_ to be below its threshold limit comprise the design space for that pressure and considered *L*_*frozen*_ value. The threshold limit, i.e., the maximum allowed temperature for the case-study formulation is usually the *T*_*g*_ or *T*_*e*_. Additionally, once the *T*_*shelf*_ and resulting *T*_*b*_ values for each pressure are known, *J*_*w*_ for any desired *L*_*frozen*_ can be calculated for the whole design space. This can be used to further optimize the process duration, by choosing the conditions within the design space that will maximize the sublimation flux.

It is important to point out that, since *R*_*p*_ has a dependence in *L*_*dried*_ the predicted *T*_*b*_ values for different *T*_*shelf*_ and *P*_*c*_ combinations will also vary according to the *L*_*dried*_ portion considered. Since *R*_*p*_ reaches its maximum value towards the end of drying, so does *T*_*b*_. Fissore et al (36) proposed the estimation of a design space including the *L*_*dried*_ as a third coordinate to account for this dynamic behaviour. In this study, we consider a static environment, i.e., one single *T*_*shelf*_ to be used throughout primary drying. To this end, all calculations are based on a critical *T*_*b*_ value using as a reference the moment when only 10% of frozen cake remains.

The use of a dynamic parameter estimation algorithm (38), manometric temperature measurement (41) and the use of a combined statistical and mechanistic approach (42) were proposed for design space estimation. However, the use of a pilot-scale or lab-scale freeze-dryer using thermocouples to monitor product temperature is still the most common tool used for the mechanistic approach. Typically, three gravimetric tests are performed for *K*_*v*_ estimation and one for *R*_*p*_, as decribed above. However, such experiments can be time consuming which increases operational costs. Specially the vial-weighting steps required for the gravimetric tests are laborious, considering that such batches usually have a few hundred vials. Additionally, poor thermocouple placement many times compromises batch monitoring if a non-expert performs this task (43).

#### Reference Method

**Fig. 1:**
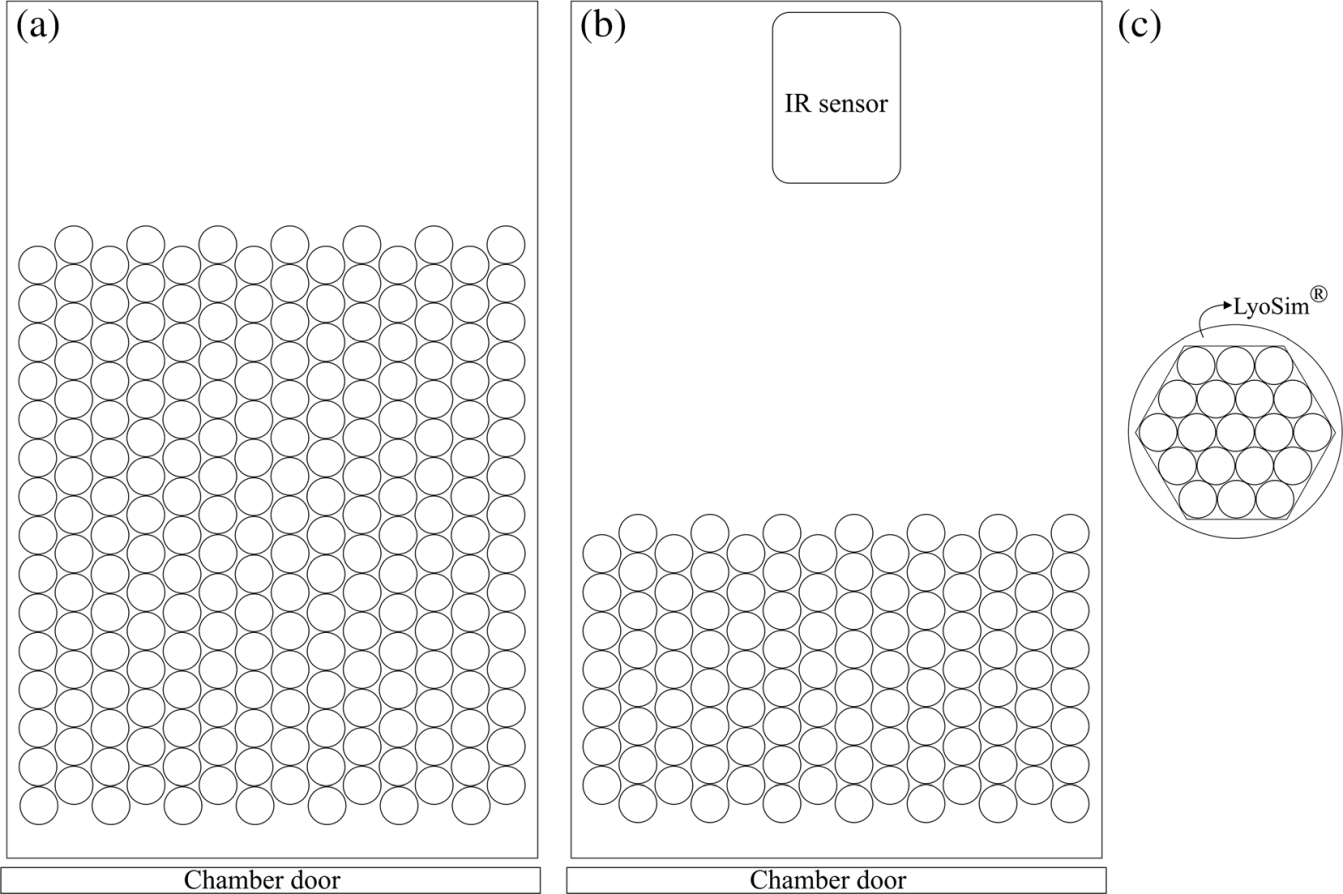
Representation of the set ups used, as seen from above, for: (**a**) the reference method, (**b**) the IR-based method (both in the REVO® freeze dryer), and (**c**) the MicroFD® method.

Design space estimations for central vial conditions using a lab scale freeze dryer (REVO®) were done. Each batch had 210 vials disposed in a hexagonal array (14 rows with 15 vials each, 156 central vials). Six thermocouples were placed in central vials for temperature monitoring. Figure 1 illustrates the batch configurations used for each of the tested methods.

#### IR-Based PAT Method

The use of an infrared sensor to monitor batches with up to 157 vials in the REVO® freeze-dryer was verified previously (33). Inspired by a previous study (44) on the measurement of thermal profiles in vials in different batch positions, an extrapolation was done. This aimed to address the IR sensor main limitation (33) when monitoring freeze-drying batches, i.e., its field of view. In that study (44), first row vials in a more shielded position in the hexagonal array configuration were shown to present a closer behaviour to central vials, although they are still different. The use of a hexagonal array permitted better batch representativeness in IR-monitored batches (33). The first row vials, in the rear of the chamber, that were slightly shielded by their side vials in this array configuration, were regarded as representative of central vials. They have only five neighbouring vials, instead of six, as a common definition of central vials. Nonetheless, this approximation allowed the estimation of model parameters, *K*_*v*_ and *R*_*p*_, for central batch conditions with good accuracy. Additionally, through monitoring of the sublimation interface temperature (*T*_*i*_) throughout primary drying, a consistent determination of the endpoint was achieved (33). The primary drying duration was determined in the same way presented by Harguindeguy & Fissore (33). The same custom MATLAB (MATLAB R2019b © 1994–2020 The MathWorks, Inc) code was used. First, a curve was fitted to the *T*_*i*_ data to allow the use of the first derivative to infer the inflection points in an automated way. The inflection point of interest is the ascending interval observed when sublimation is completed, and the heat supplied by the shelf is used as sensible heat. Since the IR sensor is non-invasive, the detection of this rising profile is much more accurate than the one observed using thermocouples and can be used to correctly infer the end of sublimation. The fitting used was a non-parametric smoothing spline, which fits a set of intersecting polynomials to the data. The function is controlled by a smoothing parameter which, the higher it is, it makes the fit smoother. The fit was calculated using MATLAB built-in *smoothingspline* function with the default parameter set (45).

Based on these findings (33), by monitoring three complete cycles using the desired formulation, the whole design space can be obtained without performing gravimetric tests. The *K*_*v*_ and *R*_*p*_ values are obtained based on the *T*_*i*_ profiles of these vials, regarded as representative of central batch ones. Eq. 5 and Eq. 6 can be used to calculate *K*_*v*_, assuming complete sublimation of the water present in the monitored vial and determining the primary drying duration by the *T*_*i*_ infrared-based method. *R*_*p*_ is directly obtained based on the *T*_*i*_ profiles of the monitored vials as previously discussed.

For these tests, 105 vials were used (14 rows with 7 or 8 vials each, 66 central vials) since some space is required to place the infrared sensor inside the chamber. Using the same equipment and settings, no significant differences were reported for the *K*_*v*_ values between these smaller IR-monitored batches and large thermocouple-monitored ones. Moreover, the effect of this sensor inside the chamber was found negligible while the shelves’ configuration was kept the same across all tests (33). Since the front row has 14 vials, this estimation is based on the profiles of the 6 more shielded vials in this row. This low number of samples could be a limitation to this method. However, the results for all tested conditions were satisfactory as reported ahead.

#### MicroFD® Method

The use of the MicroFD® with a LyoSim® offset temperature of −5°C with respect to the product temperature resulted in good batch homogeneity (46). Moreover, this offset setting was found to be a good emulator of central batch conditions in the REVO® freeze-dryer (35). This −5°C setting was also found to represent well the temperature profiles and *K*_*v*_ values of central batch vials in another freeze-drying equipment of similar scale, the LyoStar® III lyophilizer (SP Scientific, Warminster, PA, USA) (30). To determine the design space using a micro freeze-dryer, the traditional three gravimetric tests (for *K*_*v*_ estimation) and a complete primary drying cycle (for *R*_*p*_ estimation) should be performed. However, since the batch has a very small number of vials (19 in this case), the task becomes much easier, less time consuming and requires less formulation material than the usual. The MicroFD® may also be equipped with a heat flux sensor, AccuFlux®, allowing for a direct measurement of the heat flux to the product in the vials: this allows avoiding weighing the vials before and after the gravimetric test, thus further simplifying the experiments. This tool, however, was not used in the present study to reduce the degree of freedom between the methods being compared. Thus, *K*_*v*_ was estimated gravimetrically and *R*_*p*_ based on Eqs. 1 and 2.

### Products and Vials

To determine the design space for amorphous and crystalline systems, tests were carried out using 5% sucrose and 5% mannitol aqueous solutions. Both sugars were purchased from Sigma Aldrich (≥99.5%) and used as received. Solutions were processed in 6R tubing vials (Schott Pharmaceutical Packaging, Inc., Lebanon, PA, USA) using a 3 mL fill volume, resulting in a 11 mm cake height.

All vials were placed directly onto the shelf and were partially stoppered using an igloo stopper (NovaPure Chlorobutyl Igloo Stoppers, West Pharma, Exton, PA, USA) after filling. Vials monitored using thermocouples had holders (VTH-M-0020, Millrock Technology Inc. Kingston, NY, USA) that enabled careful control and correct placement of the thermocouples used, touching the bottom of the vial (46).

### Design of Experiments

For all methods, the *K*_*v*_ estimation as a function of pressure was done at 8, 13 and 26 Pa. The *R*_*p*_ profile for sucrose solution was obtained using a − 20°C shelf temperature and 8 Pa chamber pressure setting, while for mannitol it was obtained using 0°C and 13 Pa.

The definition of the threshold temperature depends on the formulation system as mentioned, but it also depends on the tolerable final product quality. For sucrose 5%, Horn and Friess (47) reported a *T*_*g*_ of −33.7°C (7). If small micro collapses are allowed, however, a maximum product temperature value of up to −32°C could be accepted (37). In this study, the threshold temperature for sucrose was defined as −33°C.

Mannitol formulations usually have a more stable cake structure, resulting in elegant final products with no observable shrinkage. Still, mannitol systems may present different polymorphs together with an amorphous phase (48). A 10% crystalline mannitol formulation presenting α-mannitol and β-mannitol polymorphs, with the former as the most abundant one, was found to have a melting temperature of −21.5°C (49). For pure amorphous mannitol, i.e., not in solution, a 13°C collapse temperature for was reported (48). Through differential scanning calorimetry (DSC) analysis, a 10% amorphous mannitol formulation was found to have two *T*_*g*_ points, one at −35°C and one −25°C (50). This formulation also showed a subsequent crystallization exotherm peak, showing the strong tendency of mannitol towards crystallization which makes it a stable cake forming agent (50, 51). Melting for this formulation was observed near 0°C, which was attributed to ice melting. Since lyophilization is based on operating below the water triple point, this melting transition should not affect freeze-dried formulations. Still, lyophilization is generally used for heat sensitive molecules, for this reason a threshold value of −15°C was chosen for the design space calculation of the 5% mannitol solution.

### Statistical Analysis

Statistical analysis was used only to compare the *K*_*v*_ values obtained through the proposed tools with the reference approach (gravimetric tests in the REVO® freeze-dryer, using thermocouple measurements). The evaluations done always compared the group values in pairs. For example, the *K*_*v*_ values at 13 Pa obtained in the micro freeze-dryer versus the ones obtained in the REVO® at the same pressure. This way, first, a normality test was performed on each group of results and then they were compared using a Student’s t test (52). The t-tests done were two tailed, two-sample (independent) t-tests assuming an unknown variance. A 99% confidence interval was used for both the normality tests and the t-tests.

## Results

### Model Parameters for Design Space Calculation

To evaluate the applicability of the proposed tools for design space estimation, first, their ability to properly obtain the model parameters must be verified. These critical model parameters are the *K*_*v*_ values at different pressures and *R*_*p*_ values for each of the tested solutions. If the values found using the proposed devices are comparable to the ones obtained through the reference method, so should be the resulting design spaces.

**Fig. 2:**
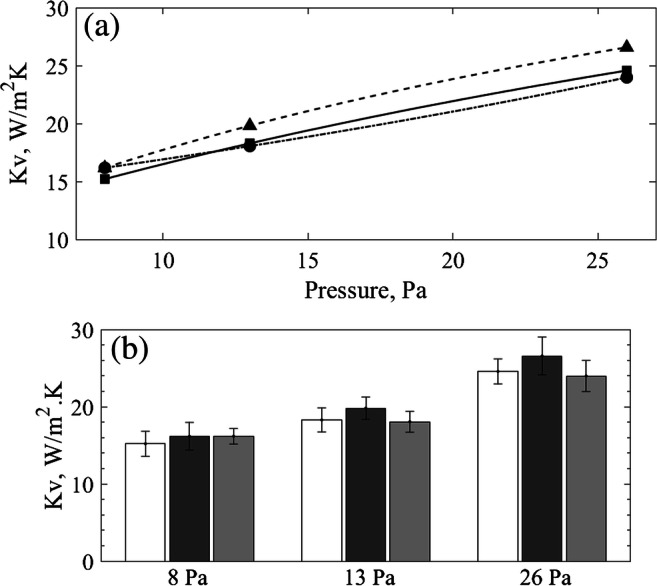
(**a**) *K*_*v*_ values with curve fit using Eq. 7 for the standard method (−■), the IR-based method (--▲) and the MicroFD® (−.●). (**b**) Bar chart for *K*_*v*_ values for the standard method (white), the IR-based method (dark grey) and the MicroFD® (light grey). Error bars indicate one standard deviation.

The average *K*_*v*_ values found in each system under the tested pressures were all comparable, as illustrated in Fig. 2. Compared to the reference method, the biggest differences were observed when using the IR-based method. These differences were of 6.3%, 8.4% and 8.1% for 8, 13 and 26 Pa, respectively. For the micro freeze-dryer, these differences were very low for 13 and 26 Pa, representing a 1.3% and 2.4% difference, respectively. For 8 Pa however, it reached a 6.5% difference against the reference method. Nonetheless, through statistical analysis via t-tests, the global heat exchange coefficients found using the MicroFD® and the IR-based method were not statistically different from the values obtained in the REVO® freeze-dryer using the gravimetric test (*p* > 0.01).

Once the *K*_*v*_ determination obtained through the proposed tools was deemed equivalent to the values found by the reference method, the accuracy in *R*_*p*_ determination for each tested formulation by the novel tools was examined. As investigated by Scutellà et al. (28), the cake resistance to vapor flow affects product temperature during drying. The whole purpose of calculating the design space is to ensure product temperature stays below its threshold value. Thus, correct *R*_*p*_ determination is of crucial importance. Since the *R*_*p*_ calculation is based on product temperature, the resulting profiles using the different proposed tools will be similar if the product temperature profiles are also similar. One of the most important aspects of the observed *R*_*p*_ profiles is the maximum value it reaches. This maximum will also dictate when the maximum product temperature will be observed, thus representing a critical control point for a given formulation.

**Fig. 3:**
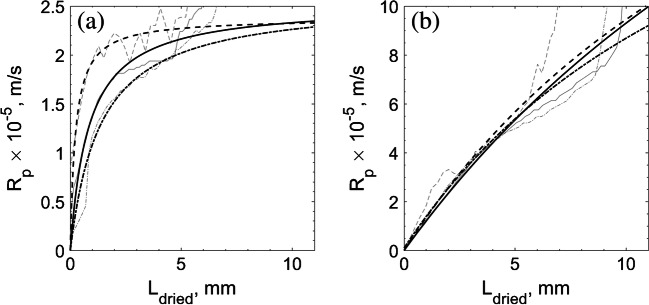
*R*_*p*_ profiles for (**a**) 5% sucrose using 8 Pa and − 20°C shelf temperature and for (**b**) mannitol 5% sucrose using 13 Pa and 0°C shelf temperature. The raw data are plotted in light grey colour while the fitted curves for the standard method (−), the IR-based method (--) and MicroFD® (-.) are in black.

Figure 3 presents the *R*_*p*_ results found for each solution. As it can be seen, for all tested tools the *R*_*p*_ profiles seem comparable, i.e., they are within the same order of magnitude and the values are quite correspondent. The temperature profiles measured by thermocouples towards the end of primary drying are not reliable because there may be a loss in contact between the sensing element and the surrounding ice (31). Moreover, unless the process is conducted by a well-trained operator, thermocouple misplacements are done, resulting in inaccurate temperature measurements (11). Additionally, even when a trained operator places the thermocouples properly, they may move during the loading process or the cake may break in such a way that it is not anymore representative of the process. Essentially, the main issue is the lack of consistency between thermocouple measurements. Many times, vials subjected to virtually the same batch conditions, present varying rising temperature profile times (31). IR thermography offers a solution to this issue since it is a non-invasive sensor and experimentally, the rising of the temperature profiles is more consistent across different vials. However, it has its own limitations as well. The IR sensor monitors the product within its field of view, which is the external product layer, in contact with the vial wall. The temperature profiles measured by the IR camera represent well the external cake layer, but they do not represent very well the last, lower inner core of the frozen product. This way, the raw *R*_*p*_ profiles observed in Fig. 3 rise before the completion of sublimation for all tested tools. That is why the fitted curves (Eq. 7) are very handy in process calculations to estimate the effective *R*_*p*_ profiles and resulting product temperatures during a process.

The durations of the process using each tested method do not directly impact the design space calculation but can also give a good clue regarding the equivalence between the tested systems. If the global heat exchange coefficient and cake resistance to vapor flow are similar between systems, so should be the overall process duration. As seen in Table I, based on the *Pi*/*Ba* curve onset and offset points, the primary drying durations were all comparable. The onset time represents the point in which drying is complete for many vials in the batch, but not yet for all of them. By the offset point, drying is complete in all vials in a batch. It is important to compare the onset and offset times together due to the large variability intrinsic to this method. These points vary according to batch size, drying conditions and equipment characteristics (31). Thus, of course they are not the *same* as the chamber volume and vacuum pump are different between the REVO® and MicroFD®. Additionally, when the IR sensor was used, the batch size, and consequently the total solvent volume, was half the size of the full REVO® batch. The time difference between the onset and offset signals derives from the batch heterogeneity, but also increases with batch size. Additionally, the *Pi*/*Ba* signal was found to start decreasing when the sublimation rate becomes smaller than a threshold value of 2 × 10^−6^ kg.s^−1^, which may vary according to the equipment and its design (31). Thus, considering the intrinsic variability of the *Pi*/*Ba* onset and offset signals, the primary drying durations observed using all tested methods may be considered to be in good agreement.

**Table I:** Primary Drying Estimated Durations in Hours Based on the *Pi*/*Ba* Onset and Offset Points

	Sucrose 5%		Mannitol 5%	
	*Pi*/*Ba* onset	*Pi*/*Ba* offset	*Pi*/*Ba* onset	*Pi*/*Ba* offset
MicroFD®	24.7	29.2	15.3	19.1
REVO-IR	25.6	33.1	15.0	16.8
REVO	28.2	33.2	16.9	20.1

### Calculation of the Design Space

**Fig. 4:**
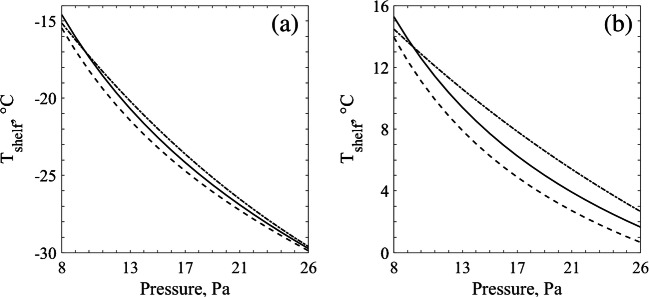
Design spaces for the last 10% of frozen cake obtained through the novel tools compared with the one obtained through the standard method. Lines plotted for the standard method (−), the IR-based method (--) and the MicroFD® (−.). (**a**) Results for sucrose 5% and (**b**) for mannitol 5%.

The design space calculation depends heavily on the *K*_*v*_ and *R*_*p*_ values found for a given product, vials used and batch configuration. Since these parameters presented a good equivalence across the systems, a similar behaviour is expected for the resulting design spaces. Figure 4 presents the upper limit of shelf temperature and chamber pressure settings for the last 10% of frozen cake obtained through all the tested methods. As it can be seen, the resulting design spaces for sucrose are practically *the same* whether they were calculated based on the proposed tools or the reference method. For mannitol, some small differences were observed in the design spaces. In the case of the MicroFD®, the lower *R*_*p*_ profile for mannitol compared to the reference method resulted in slightly higher usable *T*_*shelf*_ settings, which was more evident for higher pressures, where the MicroFD® *K*_*v*_ was smaller than the reference one.

Simulating in silico the freeze-drying process until the last 10% of cake, as described in materials & methods, the product bottom temperatures (*T*_*b*_) were calculated. With this *T*_*b*_ and Eqs. 1 and 4, *J*_*w*_ curves for different *T*_*shelf*_ and *P*_*c*_ values (since it will change the *K*_*v*_) were calculated. This information coupled with the design space allows further optimization towards reducing the required primary drying time when higher sublimation rates are chosen.

**Fig. 5:**
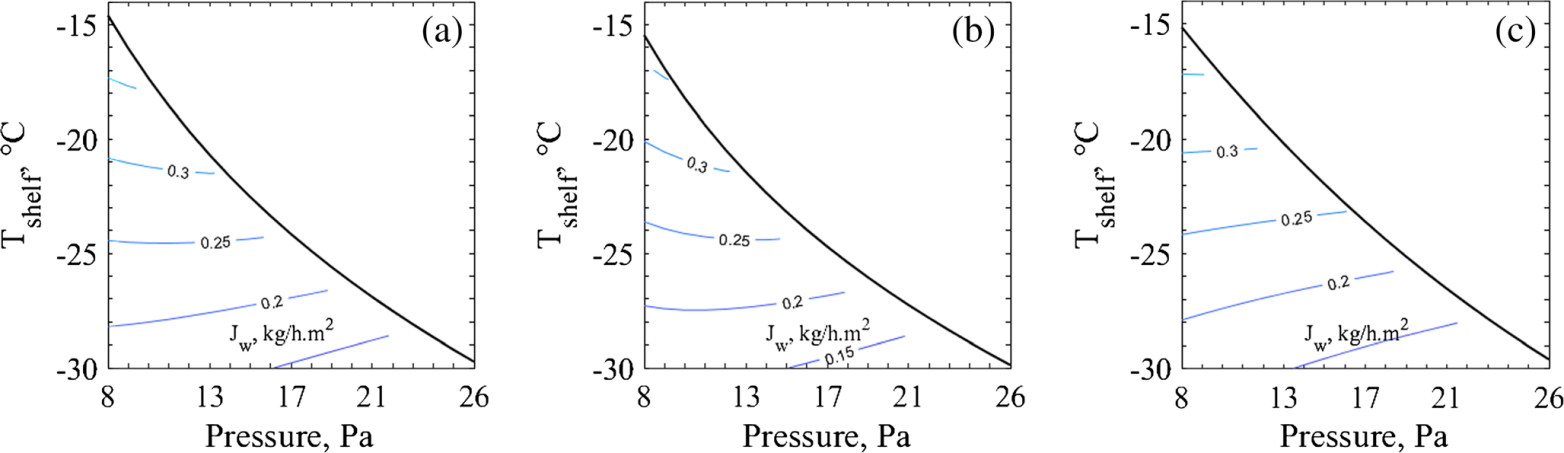
Design space for 5% sucrose considering coupled with the respective *J*_*w*_ contour plots. Obtained through (**a**) the MicroFD® (**b**) the IR-based method and (**c**) the standard method.

**Fig. 6:**
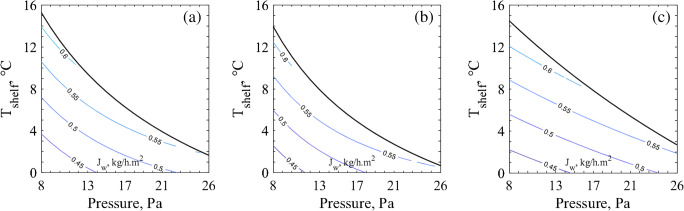
Design space for 5% mannitol considering coupled with the respective *J*_*w*_ contour plots. Obtained through (**a**) the MicroFD® (**b**) the IR-based method and (**c**) the standard method.

As presented in Fig. 5, the direction towards higher sublimation rates for sucrose is along the lower pressures, which is in accordance with previously reported results (18, 36, 37). When lower pressures are used, *K*_*v*_ also decreases allowing higher shelf temperatures. This increased shelf temperature setting provides more heat for sublimation without compromising the cake structure. According to these results, the optimal direction for choosing the operating conditions is towards the left and the top. However, in Fig. 6, the sublimation flux curve behaviour was different, more convex, making optimization direction to be towards higher pressures. This means that the increase in *K*_*v*_ when operating at higher pressures contributes more to the sublimation rate than the decrease in vapor pressure when operating at low chamber pressure settings. At first glance, this may seem different from previously reported sublimation flux contour plots (18, 36, 37); however, it is not quite the case. Taking a closer look on some previously published *J*_*w*_ contour plots (18, 36, 37), it is clear that the curves have a concave profile at lower *T*_*shelf*_ values which increasingly becomes less concave with higher *T*_*shelf*_ values, until it finally becomes convex. This matter did not affect the optimization direction of those design spaces because the change in the profile profile only occurred around the *T*_*shelf*_ upper limit. The same can be observed on Fig. 5. However, for mannitol, the *T*_*shelf*_ upper limit in Fig. 6 is roughly 15°C higher than previously calculated (18) due to the higher threshold temperature chosen in this present study. This explains the apparent differences observed in the *J*_*w*_ contour plots, having a convex profile.

It is important, however, to remember that the design spaces presented here are built for central batch conditions. Since edge vials would heat up more due to less shielding, in this case it is advisable to operate within a safety margin. In fact, it is always advisable to operate under a safety margin to ensure product quality (53). For central batch conditions, a safety margin of 2°C was proposed, considering only the variability in vial dimensions, which affects the vial *K*_*v*_. Moreover, the authors suggested that the safety margin for vials subjected to edge effects could be in the same order of magnitude of the 2°C reported value (16). Another alternative is to use the proposed tools to determine the design space considering edge vials. The proposed tools in this study can be used to determine the design space based on edge vials simply by changing the settings used for *K*_*v*_ determination. However, choosing operating conditions aiming to preserve product quality in edge vials is not practical in industrial applications. In such cases, batches are very large and edge vials comprise a small percentage of the whole batch. Since edge vials receive much more heating from the chamber walls than central vials, substantially lower shelf temperatures should be used. As seen from the *J*_*w*_ results, this would increase greatly the total required drying time, representing a big increase in processing costs just to preserve a very small percentage of the batch. Longer cycles with lower shelf temperatures can ensure product quality for the whole batch, but also mean less batches produced per year, which increases the capital costs per cycle. The final decision on how to design a cycle will be based on what delivers a quality product at the fairest price to the patients.

Comparing the behaviours of the *T*_*shelf*_ and *P*_*c*_ upper limit line together with the *J*_*w*_, it can be appreciated that the MicroFD**®** tended to have a more linear behaviour than the observed ones for the IR-based method and the reference approach (both in the REVO). This is simply a direct reflection of the behaviour of the fitted curve to *K*_*v*_, also more linear and it does not have any relevant physical meaning. In fact, the variation between the resulting design spaces using the novel tools in comparison to the reference method is irrelevant from the practical point of view, because it is advisable to operate with a safety margin as above-mentioned.

### Final Considerations on the Design Spaces Obtained

**Fig. 7:**
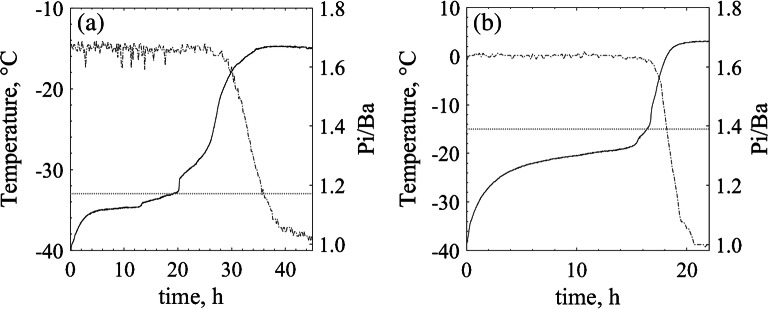
Product temperature (−) and pressure ratio signal (−.) during primary drying for the standard method (REVO**®** full batch monitored through thermocouples) using (**a**) sucrose 5% at −20° and 8 Pa. (**b**) mannitol 5% at 0°C and 13 Pa. The horizontal (..) grey lines show the threshold temperature for each solution.

To verify the applicability of the obtained design spaces, product temperature must stay below the threshold value when operating under these conditions for a REVO**®** full batch, with 210 vials. Considering all previous similarities in *K*_*v*_ and *R*_*p*_ between the different methods, this is expected to happen. As follows, Fig. 7 shows the temperature profiles and pressure ratios (*Pi*/*Ba*) observed through a complete primary drying cycle for both tested solutions. The tests presented were the same ones used to determine the *R*_*p*_ profiles for the reference method. For both products, the conditions chosen are below the *T*_*shelf*_ and *P*_*c*_ pairs upper limit by a margin and, so do the resulting temperature profiles.

As it can be observed, product temperature was kept well below the defined threshold values. As explained above, thermocouple measurements are not reliable towards the end of primary drying. Thus, if by the end of primary, the temperature profiles are above the threshold value, that may not represent product jeopardy. In freeze drying, as in many other processes, several factors influence the final product quality, this way, a holistic analysis of the results is preferred over a reductionist one, which relies on just one sensor or attribute to evaluate and develop a cycle.

## Discussion

The heat and mass transfer coefficient results are in accordance with previous findings. The same IR sensor was previously applied to the same batch configuration of 6R vials using 8 Pa and − 20°C as operating conditions. In that experiment, *K*_*v*_ was calculated gravimetrically using the temperature profile provided by the infrared sensor. The resulting *K*_*v*_ found in that study was 16.7 ± 2.3 W/m^2^K (33). In this present study, the non-gravimetric *K*_*v*_ estimation resulted in 16.2 ± 1.8 W/m^2^K. For the MicroFD®, using the −5°C offset setting for the LyoSim®, *K*_*v*_ values in the MicroFD® were found correspondent to REVO® central batch ones (35). The observed *R*_*p*_ values found in this study were also in good agreement with previously reported values (18, 28).

It can be appreciated that the resulting design spaces obtained through the different tested tools and approaches are all in good agreement. It is important to remember, however, that these results consider a static design space. Thus, only one shelf temperature setting was used, considering the last 10% of frozen cake as a critical control point in this process. Still, the design space is a result of the process parameters *K*_*v*_ and *R*_*p*_. Since there was a good equivalence between the proposed tools and the reference method, the results suggest these tools could be also used considering different percentages of remaining frozen cake. This would allow the development of a dynamic design space, taking advantage of the lower *R*_*p*_values in the beginning of drying to use higher *T*_*shelf*_ values and decrease the required drying time.

Furthermore, to scale the design space obtained using the reference method up to an industrial freeze dryer, only one extra gravimetric test may be sufficient as described in Fissore et al. (18). One test is enough in fact to determine the *a*_*Kv*_ coefficient from Eq. 7, the only one with a relevant dependence on the equipment, given that the fit was already done in a lab-scale or pilot-scale freeze dryer. The *R*_*p*_ should also be obtained for the industrial equipment, but again just one test would be enough for a given formulation. This scale-up method can also be analogously used for the proposed novel tools, since such a good agreement was found between the tested methods. Regarding chocked flow, in lab scale this is typically less usual due to the equipment design (18). Still, it can be an issue when high sublimation rates are used for industrial scale freeze-dryers. To address this, the industrial equipment should be tested at full capacity and different pressures as described in Patel et al. (12). This should be done just once and it can be used for all future process design for that  piece of equipment.

## Conclusions

Both alternative methods investigated in this paper for design space estimation present advantages and limitations. The non-gravimetric *K*_*v*_ determination obtained using the IR camera is favourable in terms of not having to weigh numerous vials before and after primary drying. Additionally, with just a complete run *K*_*v*_ and *R*_*p*_ profiles can be promptly obtained. However, it is time consuming since the entire drying cycle must be performed at each tested pressure. Is it important to point out, as mentioned in the introduction, that there are other methods which allow a non-gravimetric estimation of *K*_*v*_ and the estimation of *R*_*p*_. Many are based on the Pressure Rise Test; some rely on Tuneable Diode Laser Absorption Spectroscopy or use a heat flux sensor, for instance. These methods also present advantages of making *K*_*v*_ and *R*_*p*_ determination less laborious. Still, another issue to be taken into consideration for the IR-based method is the cost as each run requires the use of the actual product. This way, this method is recommended mostly when the tested product is not prohibitively expensive, and when the time required to prepare the batch, to load/unload the vials and to defrost the condenser is not a concern.

The MicroFD® once again showed its practicality and applicability. *K*_*v*_ can be estimated by the traditional method, i.e., gravimetrically without much hassle since only 19 6R vials are needed. The MicroFD® is equipped with a heat-flux sensor, AccuFlux® which also allows the non-gravimetric determination of the *K*_*v*_, although this sensor was not used in this study. Additionally, only a small amount of actual product is needed to obtain the *R*_*p*_ profile. This is recommended when dealing with very expensive materials or with new formulations that need to be further studied, saving time for batch preparation.

This study presented only the use of these tools applied to the design space estimation for central batch conditions. Additionally, the study considered only a static environment and did not include uncertainties, derived from batch variability, into the design space. All these non-explored approaches may be included in future research. Additionally, future work could explore a combined alternative of a non-gravimetric *K*_*v*_ estimation in a very small scale in a MicroFD®. This way all advantages of the proposed novel tools would be retained while removing the limitations of each approach.

## List of Symbols

*A*: parameter used to model the dependence of *R*_*p*_ on *L*_*dried*_, s^−1.^
${a}_{K_v}$: parameter used to model the dependence of *K*_*v*_ on *P*_*C*_, W m^−2^K^−1^
*A*_*v*_: vial bottom area, m^2^
*B*: parameter used to model the dependence of *R*_*p*_ on *L*_*dried*_, m^−1^
${b}_{K_v}$: parameter used to model the dependence of *K*_*v*_ on *P*_*C*_, W m^−2^K^−1^*Pa*^−1^
${c}_{K_v}$: parameter used to model the dependence of *K*_*v*_ on *P*_*C*_, *Pa*^−1^
∆*H*_s_: heat of sublimation, J kg^−1^
*J*_*q*_: heat flux, W m^−2^
*J*_*w*_: mass flux, kg s^−1^m^−2^
*k*_*frozen*_: ice thermal conductivity, W m^−1^K^−1^
*K*_*v*_: heat transfer coefficient, W m^−2^K^−1^
*L*_*dried*_: dried cake thickness, m
*L*_*frozen*_: frozen cake thickness, m
∆*m*: mass change, kg
*p*_*w*, *c*_: water vapor partial pressure in the drying chamber, Pa
*p*_*w*, *i*_: water vapor partial pressure at the sublimation interface, Pa
*P*_*C*_: chamber pressure, Pa
*Pi*/*Ba*: thermal conductivity and capacitance gauges pressure ratio, −
*Q*: heat received by the product, J
*R*_*p*_: cake resistance to vapor flow, m s^−1^
*R*_*p*, 0_: parameter used to model the dependence of *R*_*p*_ on *L*_*dried*_, m s^−1^
*T*_*b*_: product temperature at the vial bottom, K
*t*_*d*_: gravimetric test duration, s
*T*_*i*_: temperature at the sublimation interface, K
*T*_*shelf*_: shelf temperature, K
